# Editorial: Exploring machine learning applications in visceral surgery

**DOI:** 10.3389/fsurg.2025.1623356

**Published:** 2025-05-21

**Authors:** Marialuisa Lugaresi, Christof Mittermair, Gabriel Sandblom

**Affiliations:** ^1^ Department of Medical and Surgical Sciences, University of Bologna, GVM Care & Research, Bologna, Italy; ^2^ Department of Surgery, Saint John of God Hospital, Teaching Hospital of the Paracelsus Medical University Salzburg, Salzburg, Austria; ^3^ Department of Clinical Science and Education Södersjukhuset, Department of Surgery, Karolinska Institutet, Södersjukhuset, Stockholm, Sweden

**Keywords:** machine learning, deep learning algorithms, predictive modeling, decision support, personalized treatment, risk stratification, computer-assisted surgery, visceral surgery

**Editorial on the Research Topic**
Exploring machine learning applications in visceral surgery

The landscape of visceral surgery is poised at the edge of a profound transformation. Artificial intelligence, and in particular machine learning (ML), is no longer a distant technological promise but a rapidly unfolding reality within surgical practice. Across every phase of surgery, from preoperative assessment to intraoperative decision-making and postoperative recovery, ML offers unprecedented opportunities to refine, personalize, and elevate the quality of care.

Despite the proliferation of robotic systems, high-resolution imaging, and digital infrastructures within modern operating rooms, the true clinical integration of ML remains in its infancy. Yet the momentum is undeniable. ML algorithms, capable of digesting immense volumes of complex, heterogeneous clinical data, are beginning to reveal insights beyond human perception. They do not merely automate analysis; they transform it, identifying hidden patterns, predicting outcomes with remarkable accuracy, and supporting decision-making in ways that promise to redefine surgical precision and patient safety.

The contributions featured in this issue offer a compelling snapshot of this revolution in motion. In one study, Avram et al. harnessed a random forest (RF) model to predict the presence of colorectal polyps, recognized precursors to cancer. Drawing from common clinical parameters, body mass index, glucose, hemoglobin, cholesterol, liver enzymes, the model achieved impressive predictive performance, with area under the curve (AUC) scores of 0.82 and 0.79 in internal and external validation, respectively. Compared with traditional generalized linear models and support vector machines, the RF model captured complex nonlinear relationships, enhancing early risk stratification and opening new avenues for cancer prevention.

Elsewhere, Wen and collaborators tackled the critical challenge of predicting hospital stay duration after colorectal surgery. Training ten distinct ML models on 40 clinical variables from 83 patients, they ultimately found that logistic regression, after thoughtful feature selection with Lasso regression, outperformed more complex approaches, achieving an AUC of 0.99. Intriguingly, simple measures such as distance walked on postoperative day three emerged as dominant predictors, confirmed by Shapley additive explanations (SHAP). This insight underscores a broader truth: even within the world of sophisticated algorithms, meaningful clinical features often remain rooted in the human experience of recovery.

Postoperative frailty, particularly in elderly patients undergoing enterostomy, represents another frontier where ML is beginning to show its value. Zhang et al. developed predictive models across a multicenter cohort of 362 patients, with extreme gradient boosting (XGBoost) consistently outperforming logistic regression, Bayesian classifiers, and support vector machines across a range of performance metrics. Early identification of frailty risk can empower clinicians to implement tailored interventions, potentially preventing complications and shortening recovery times.

ML's impact is not limited to colorectal and enterostomy surgery. In high-stakes cardiovascular operations, such as type A aortic dissection repair, Li et al. demonstrated that RF models could predict postoperative gastrointestinal bleeding with an AUC of 0.93, outperforming k-nearest neighbors, support vector machines, and decision trees. SHAP analysis illuminated key risk factors, including ventilation duration and transfusion requirements, offering actionable insights for perioperative management.

In the field of gastric cancer, Chung et al. leveraged ML to predict duodenal stump leakage, a rare but devastating complication, analyzing data from over 1,100 patients. Here again, ensemble methods such as XGBoost and RF led the pack, highlighting the predictive power of data-driven approaches even for rare outcomes. Notably, predictive accuracy improved dynamically over time, peaking at postoperative day seven, emphasizing the potential of continuous, real-time clinical monitoring.

These individual studies are framed within a broader context by Hossain et al. comprehensive review of ML applications across the surgical continuum. Preoperatively, ML models have already outperformed traditional risk scores in predicting surgical risks and complexities. Intraoperatively, deep learning models such as GoNoGoNet and DeepCVS have begun to guide surgeons in distinguishing safe from hazardous zones, enhancing precision in laparoscopic procedures. Postoperatively, ML is advancing both complication prediction and surgical education. Automated skill assessment platforms, integrating computer vision and ML, are emerging as powerful tools for objective feedback, promising a future where surgical training is continuously informed by real-time analytics.

Yet, amid these exciting advances, significant challenges remain. Clinical data, the lifeblood of ML, is often incomplete, inconsistent, and non-standardized. Models trained on such data risk being brittle, less generalizable, and vulnerable to biases. Moreover, the black-box nature of many ML algorithms raises critical questions around interpretability and trust. Without transparency, even the most accurate model may struggle for clinical acceptance.

The solution lies not only in better algorithms but in closer collaboration. Surgeons, data scientists, engineers, and regulatory bodies must work hand-in-hand to design ML tools that are not only powerful but also explainable, ethical, and seamlessly integrated into clinical workflows. Tools like SHAP that demystify model outputs are steps in the right direction, but broader cultural shifts are needed, toward data literacy among clinicians, transparent model development, and rigorous, independent validation.

Ethical considerations loom large as well. Data privacy, algorithmic fairness, and accountability for ML-driven decisions must be addressed with the same rigor applied to any other medical intervention. Only through proactive governance and cross-disciplinary dialogue can the full potential of ML be realized in an equitable, patient-centered way.

Looking ahead, the question is not whether ML will transform visceral surgery, but how and how quickly. The studies in this issue demonstrate that the future is not a distant prospect; it is already taking shape today. With thoughtful integration, continuous learning, and a steadfast commitment to patient welfare, ML can evolve from an experimental innovation into a trusted partner in the surgical journey.

In doing so, it promises not only to enhance outcomes but to fundamentally reimagine the art and science of surgery itself.

